# Understanding the Interplay between Insulin Resistance and Cognitive Function: A Longitudinal study conducted in the Urban Cohort, Southern India

**DOI:** 10.1002/alz.092162

**Published:** 2025-01-03

**Authors:** Anjana J Menon, Monisha S, Thomas Gregor Issac

**Affiliations:** ^1^ Centre for Brain Research, Bengaluru, Karnataka India; ^2^ Centre for Brain Research, Indian Institute of Science, Bengaluru, Karnataka India; ^3^ Centre for Brain Research, Indian Institute of Science, Bangalore, Karnataka India

## Abstract

**Background:**

Recent epidemiological studies have shown that diabetes can have negative effects on cognitive function, and insulin resistance (IR) acts as a key factor in this association. IR can impede glucose uptake in brain, leading to energy deficits and toxic protein accumulation which may cause cognitive decline. Type 3 diabetes mellitus (T3DM), or AD type of diabetes is caused due to impaired insulin signalling restricted to brain regions, resulting in memory decline. This study therefore aims to explore the link between IR and cognitive dysfunction, which will help in understanding the role of IR as a potential risk factor of cognitive impairment.

**Method:**

This study utilized 1007 participants aged 45 years and above, recruited by CBR‐Tata Longitudinal Study of Ageing (TLSA), which is a community‐based urban longitudinal study cohort. 569 participants were with IR, and 438 participants were healthy. Addenbrooke’s Cognitive Examination (ACE III) was utilised to assess cognitive performance of the participants and IR was measured using Homeostatic Model Assessment‐insulin resistance (HOMA‐IR). Mann Whitney U test was performed to compare between ACE scores and IR status. Further, Generalized Linear Model (GLM) analysis was performed between cognitive variables and IR status after adjusting for covariates.

**Result:**

Mann Whitney U test showed a statistically significant association between IR status and ACE scores in ACE total, ACE attention and ACE visuospatial. GLM analysis revealed a significant negative correlation between IR status and performance in attention (B = ‐0.271, 95% CI (‐0.468, ‐0.073), p<0.05), as well as in visuospatial tasks (B = ‐0.258, 95% CI (‐0.494, ‐0.022), p<0.05). After adjusting for confounders such as age, education, and inflammatory marker like CRP, results showed that ACE attention scores (B = ‐0.396, 95% CI (‐0.396, ‐0.008), p<0.05) were significantly lower in participants with IR.

**Conclusion:**

According to the findings of our study, insulin resistance was associated with poor cognitive outcome in the attention domain. This finding could be attributed to the increased sensitivity of prefrontal cortex to altered insulin levels. Thus, considering the association between IR and cognition, it is important to devise strategies that can help in detecting and treating IR at an early stage to prevent cognitive impairment.

